# Deep Learning in Pancreatic Tissue: Identification of Anatomical Structures, Pancreatic Intraepithelial Neoplasia, and Ductal Adenocarcinoma

**DOI:** 10.3390/ijms22105385

**Published:** 2021-05-20

**Authors:** Mark Kriegsmann, Katharina Kriegsmann, Georg Steinbuss, Christiane Zgorzelski, Anne Kraft, Matthias M. Gaida

**Affiliations:** 1 Institute of Pathology, University of Heidelberg, 69120 Heidelberg, Germany; christiane.zgorzelski@med.uni-heidelberg.de; 2 Department of Hematology, Oncology and Rheumatology, University of Heidelberg, 69120 Heidelberg, Germany; katharina.kriegsmann@med.uni-heidelberg.de (K.K.); georg.steinbuss@med.uni-heidelberg.de (G.S.); 3 Institute of Pathology, University Medical Center Mainz, JGU-Mainz, 55131 Mainz, Germany; matthias.gaida@unimedizin-mainz.de; 4 Research Center for Immunotherapy, University Medical Center Mainz, JGU-Mainz, 55131 Mainz, Germany; 5 Joint Unit Immunopathology, Institute of Pathology, University Medical Center, JGU-Mainz and TRON, Translational Oncology at the University Medical Center, JGU-Mainz, 55131 Mainz, Germany

**Keywords:** pancreatic cancer, convolutional neuronal networks, artificial intelligence, deep learning

## Abstract

Identification of pancreatic ductal adenocarcinoma (PDAC) and precursor lesions in histological tissue slides can be challenging and elaborate, especially due to tumor heterogeneity. Thus, supportive tools for the identification of anatomical and pathological tissue structures are desired. Deep learning methods recently emerged, which classify histological structures into image categories with high accuracy. However, to date, only a limited number of classes and patients have been included in histopathological studies. In this study, scanned histopathological tissue slides from tissue microarrays of PDAC patients (*n* = 201, image patches *n* = 81.165) were extracted and assigned to a training, validation, and test set. With these patches, we implemented a convolutional neuronal network, established quality control measures and a method to interpret the model, and implemented a workflow for whole tissue slides. An optimized EfficientNet algorithm achieved high accuracies that allowed automatically localizing and quantifying tissue categories including pancreatic intraepithelial neoplasia and PDAC in whole tissue slides. SmoothGrad heatmaps allowed explaining image classification results. This is the first study that utilizes deep learning for automatic identification of different anatomical tissue structures and diseases on histopathological images of pancreatic tissue specimens. The proposed approach is a valuable tool to support routine diagnostic review and pancreatic cancer research.

## 1. Introduction

Pancreatic ductal adenocarcinoma (PDAC) is the fourth leading cause of cancer-related deaths, with about 47,050 deaths and currently 57,600 estimated new cases in the United States according to the SEER database [1]. It is predicted that PDAC will rise to the second cause of cancer-related deaths in 2030 [2]. Due to non-specific symptoms and late diagnosis, only 15–20% of patients are suitable for potential curative surgery [3]. Depending on the localization of the tumor, a Whipple procedure, left pancreatectomy, or total pancreatectomy is performed [4]. The tissue of the resection specimens needs mandatory pathological evaluation to determine the extent of the adenocarcinoma, histological grade of differentiation, lymph node metastases, resection margins, preinvasive pancreatic intraepithelial neoplasia, and non-neoplastic tissue. The pathological findings will then determine further therapeutic management. To obtain a detailed picture of the tumor, the neoplastic cells have to be distinguished from benign or inflammatory lesions. This can be challenging due to the enormous inter- and intratumor heterogeneity in terms of growth pattern, cytological characteristics, and stromal properties. The heterogeneity and complex growth pattern are mainly due to an inflammatory and fibrotic microenvironment, the latter accounting for the vast majority of the tumor mass and being a hallmark of disease [5]. Microscopically, PDAC shows a predominantly glandular growth pattern with an extensive desmoplastic stroma reaction; however, also (micro-)papillary, solid nest-like, cribriform, or single-cell dissociated tumor growth can be detected [6]. Various molecular factors, such as a mesenchymal tumor phenotype, proteases, or dense infiltrates of neutrophils, are associated with a non-glandular, histologically poorly differentiated tumor growth pattern [7,8]. For PDAC, a dispersed growth pattern is typical. Here, the tumor cells often do not grow as a coherent tumor mass but rather as cellular clusters with a considerable distance from the main tumor mass, infiltrating the surrounding tissue, nerve sheaths, and vessels [9]. The precursor lesions of PDAC are called pancreatic intraepithelial neoplasia (PanIN), comparable to the adenoma-carcinoma sequence in colon cancer, where the ductal epithelial cells progress from intraductal atypical proliferations to invasive carcinoma [10]. In healthy pancreatic tissue and in chronic pancreatitis, the glandular and ductal structures are found in an organoid, lobular arrangement, whereas in PDAC, the tumor glands are arranged diffusely within the stroma, are deformed, and have incomplete lumina or show single-cell growth [11]. Chronic pancreatitis, which is a major risk factor for the development of invasive carcinoma, is histologically characterized by loss of acinar tissue, pancreatic fibrosis, and ductal changes and is often found as an accompanying component in the surrounding of PDAC [12]. Based on the very complex histological microarchitecture of PDAC, its dispersed growth, its heterogeneous microenvironment, preinvasive lesions, inflamed tissue, and sealed anatomical tissue, a common review time in diagnostics of 1–2 min per slide can be estimated [13]. Beyond diagnostic accuracy, time is also an important variable in diagnostics and will play a more important role as the overall number of specialized pathologists is currently decreasing, while the overall requirements in knowledge and specialization and the number of cases are increasing [14,15]. Thus, methods that support and facilitate morphological-based review of tissue slides and highlight critical areas for deeper investigation by expert pathologists are desirable. Digital pathology has emerged as a method not only to evaluate histopathological slides but also to support routine diagnostics and research, and to ensure quality control. Especially spatial-based tissue research needs reproducible tissue classifications. It has previously been reported that deep leaning techniques may be used to identify lymph node metastases and to classify tumor subtypes [16].

Commonly, convolutional neuronal networks (CNNs), a class of deep neural networks, are used for image classification tasks. In brief, these are made up of an input layer (image), multiple hidden layers that perform operations such as convolutions or pooling, and an output layer. Each layer is composed of neurons containing learnable weights and biases. For a detailed in-depth review of the CNN architecture and methodology, see [17].

While several reports on the identification of pancreatic cancer on computed tomography (CT) images supported by artificial intelligence exist, to our knowledge, no studies are available which show the potential of this technique in pancreatic histopathological tissue sections. There are notable differences between a well-recognized CT study using artificial intelligence in clinical imaging and the analyses at the histological level planned and performed in the present study [18]. First, clinical CT imaging captures a black-and-white image of whole body parts, which is a very different image format compared to scanned histological slides, a technology used in surgical pathology, where colored images are acquired from biopsy or resection specimens after staining. Whole slide images after scanning are about 100× larger compared to CT images. CT scans deliver images in lower magnification (anatomical structure level) compared to the technology used here, which delivers pictures at the cellular level.

In the present study, we set out to investigate if the identification of specific tissue-associated anatomical structures, pancreatic intraepithelial neoplasia, and pancreatic ductal adenocarcinoma is possible via a CNN on scanned histopathological slides.

## 2. Results

### 2.1. Patient Characteristics

Clinicopathological information was available for the tissue microarray (TMA) used in this study. The cohort consisted of 56 male and 55 female patients (n.a.: 2 patients), with a median age of 66.9 years and a median tumor size of 3.4 cm. A basic summary of the clinicopathological characteristics of the pancreatic cancer TMA cohort is provided in Table 1.

### 2.2. Image Patch Extraction

Identification of representative regions resulted in a total of 81.165 extracted 100 × 100 µm (11.125 with 395 × 395 px and 70.040 with 219 × 219 px) image patches. The number of extracted image patches is displayed in Table 2.

### 2.3. Convolutional Neuronal Network Selection and Hyperparameter Optimization

**Figure 1:**
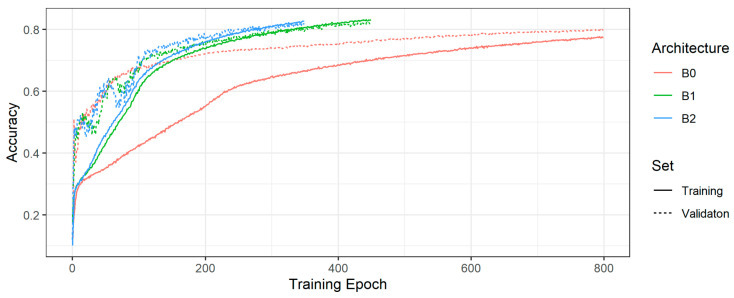
Training and validation accuracies of the model with the highest validation accuracy per EfficientNet.

Figure 1 shows the training and validation accuracies of the model with the highest validation accuracy per EfficientNet architecture type (B0, B1, B2). For the tuned models in Figure 1, Table 3 displays the chosen learning rate, batch size, and training and validation accuracies. Regarding the overall accuracies, the B1 and B2 models are almost on par, though the B2 model exhibits slightly less overfitting. Thus, we decided to use the B2 model to classify the test set.

### 2.4. Evaluation of the Test Set

**Figure 2:**
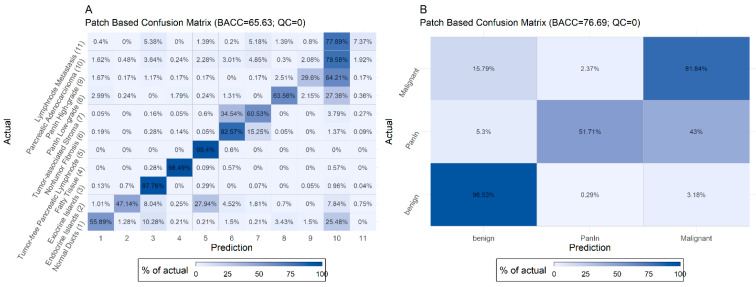
Non-aggregated (**A**) and aggregated (**B**) confusion matrices for all classes for the B2 EfficientNet model and no quality control limit.

Figure 2 displays the normalized confusion matrix of the selected B2 model in terms of the image patches for the test set. For these matrices, image patches are assigned to the predicted class which has the highest probability. We used the balanced accuracy (BACC) instead of the plain accuracy to account for the unequal distribution of classes.

The model shows a high recall rate of >90% for exocrine parenchyma, fatty tissue, tumor-free, and lymph nodes. Misclassified cases mainly (>5%) occurred (i) in the group of normal pancreatic ducts, which were classified as exocrine islands or adenocarcinoma, (ii) in the group of endocrine islands, which were classified as exocrine parenchyma, (iii) in the group of non-tumor fibrosis, which was classified as tumor-associated stroma, (iv) in the group of tumor-associated stroma, which was classified as non-tumor fibrosis or adenocarcinoma, (v) in the group of PanIN low- and high-grade and adenocarcinoma, which were mainly misclassified within these three groups and normal ducts, and (vi) in the group of lymph node metastases, which were classified as pancreatic adenocarcinoma. Many of these misclassifications can be explained (see Discussion).

### 2.5. Introduction of Quality Control Threshold

**Figure 3:**
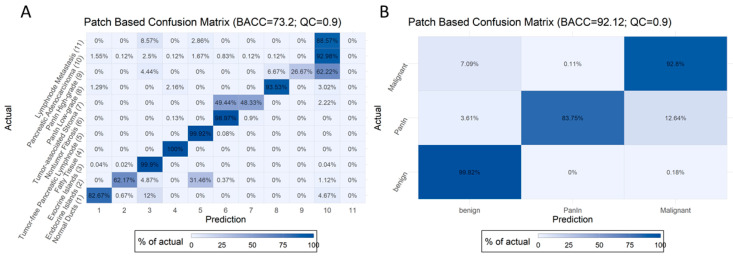
Non-aggregated (**A**) and aggregated (**B**) confusion matrices for all classes for the B2 EfficientNet model and quality control limit of 0.9.

Figure 3 features the optimal BACC and quality control threshold on our data at the image patch level (BACC = 73% for non-aggregated and 92% for aggregated data). Any patch with a predicted probability (in terms of the highest prediction probability) of less than the patch-based quality control (PQC) threshold was filtered out. Image patch confusion matrices for QC of 0.5, 0.6, 0.7, 0.8, and 0.9 can be found in Figure S1, which show an improved BACC at the patch level from 67 to 73% in non-aggregated and from 79 to 92% in aggregated data in the test set.

As many images may show more than one class (e.g., normal pancreatic ducts surrounded by exocrine parenchyma, or endocrine islands within exocrine parenchyma), a non-aggregated accuracy may actually underestimate the overall power of our model. Thus, we aggregated all benign, both PanIN, and all malignant classes into three categories: benign, PanIN, and malignant. The highest aggregated BACC of our best model was 92.12%, with a PQC threshold of 0.9 for the test set. However, it should be noted that a high proportion of cases (53.59%) are excluded in this case, as they do not pass the PQC (Table 4).

### 2.6. Explainability of the Model

**Figure 4:**
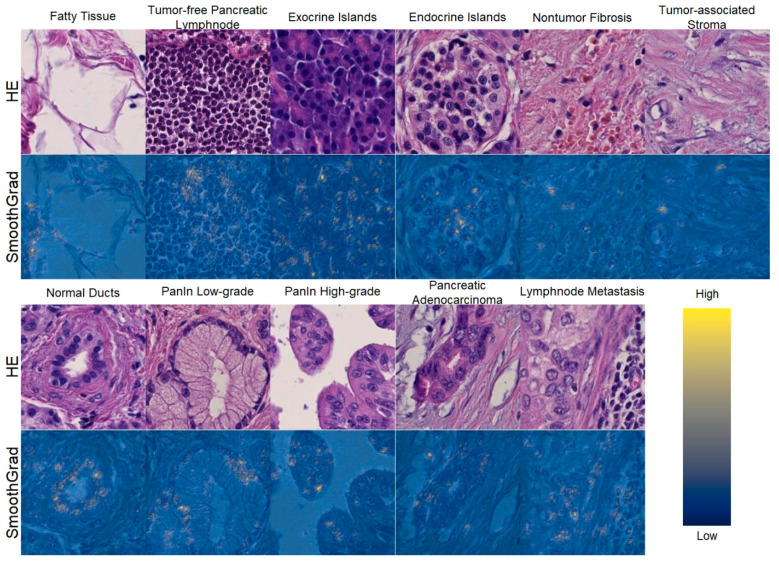
SmoothGrad heatmaps of exemplary image patches that have been classified correctly and highlight cellular structures. For each class, the upper plot shows the original image patch, while the lower plot shows the patch overlaid with the SmothGrad heatmap in respect to the class of the image patch. High SmoothGrad activity scores can be seen in areas overlaid with cells or nuclei. This confirms that the algorithm classified the image patches based on cellular morphology. Scale: each image 100 × 100 µm (219 × 219 px).

We observed high class-specific activity in cellular and non-cellular structures using SmoothGrad (Figure 4). For example, in normal non-dysplastic pancreatic ducts, nuclei are particularly important for the algorithm to detect ducts and to classify the ducts as normal/non-dysplastic. Thus, we conclude that our model predicts the respective class based on morphological features and not on technical characteristics.

### 2.7. Application on Whole Slides

**Figure 5:**
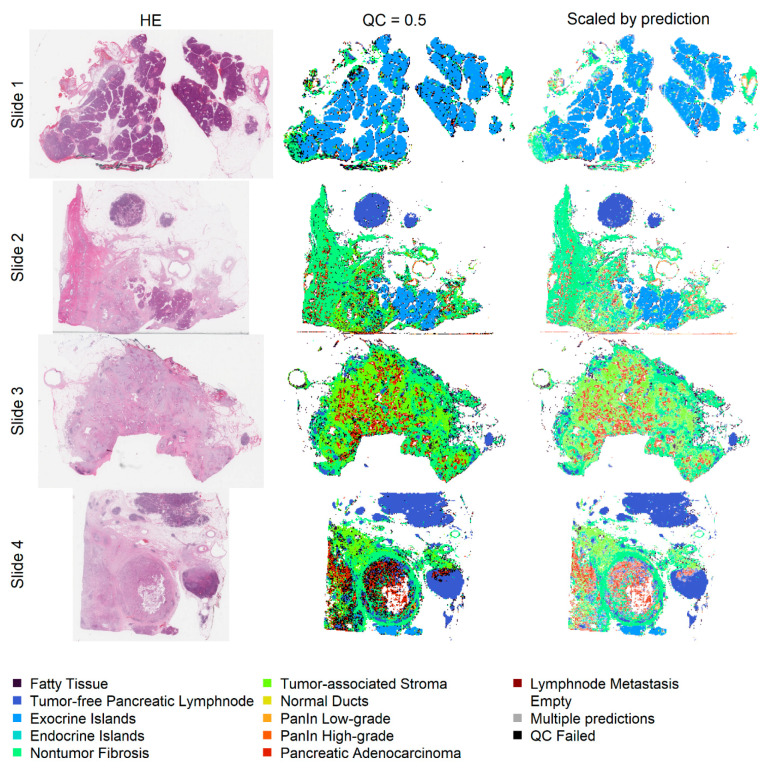
The first column shows the original slides, the second column shows the patch classification heatmaps with a QC of 0.5, and in the last column, the patch color is mixed with white depending on the prediction probability (1 results in no mixing with white). Non-tumor pancreatic tissue (first row), areas with a high content of pancreatic adenocarcinoma (second to fourth rows), and lymph node metastases (fourth row) can be easily identified.

Prediction heatmaps show the localization of automatically classified image patches within whole slides after eliminating image patches with a low optical density in order to save computational resources. The different anatomical structures can easily be identified. Additionally, the identification of areas with a high tumor cell content is facilitated (Figure 5). The number of classified image patches is displayed in Table S1.

## 3. Discussion

The aim of the present study was to generate a deep learning approach which can distinguish pancreatic cancer tissue into distinct pathological and anatomical structures. For this, we classified 11 different tissue structures that occur within the tissue of pancreatic resection specimens by a CNN with high accuracy. We further established a method to enhance the explainability and interpretability for single image patches and, moreover, a technique to automatically visualize different anatomical tissue structures on whole slides.

Deep learning techniques and particularly CNNs have been previously applied to different types of scanned histopathological images [19,20,21,22]. Application of these techniques to histopathological slides is particularly complex, due to the enormous file size, which is about 100 times larger compared to clinical CT scans. On the other hand, these large datasets contain large amounts of information, which can be used to differentiate various tissue types, disease patterns, and even tissue-based molecular alterations. In contrast to other histopathological studies, we not only included singular or a few but more than 10 structure classes in our algorithm [23,24]. Beyond the technical side, our study is, to our knowledge, the first in which deep learning techniques are applied to a large cohort of pancreatic tissue specimens and thus is of potential interest for clinical pathologists and researchers in the field of PDAC.

Technically, our study applies an up-to-date workflow regarding the study design, the image patch size, the use of a recent model architecture, the use of quality control measures, the application of a method to explain and interpret the model, and the application of the generated tools on whole tissue slides.

To obtain the highest accuracy, we used a training, a validation, and a test set, where all images from each individual patient were grouped only in one cohort, in order to ensure the most reliable results. The best results were obtained when using three datasets, compared to investigations with limited numbers of patients, which only use two sets for training and validation [23]. This is suboptimal, as very high accuracy values may be achieved by tuning the algorithm to the training and validation sets [25].

Our image patch size of 100 × 100 µm that was chosen arbitrarily, but it is in the range of the reported and well-accepted image patch sizes of 16 × 16 px and 800 × 800 px [24]. Some of the differences can be explained by the fact that investigators have previously extracted images at 200× magnification [21,26]. However, most studies have extracted the image patches at 400× magnification, as in our study, to ensure the highest resolution of picture information [27,28]. With about 81.165 extracted image patches from 201 patients, our study belongs to the largest and can be compared with other studies on histopathological images [29].

To obtain the highest accuracy, we trained our model with the EfficientNet framework because it has achieved high top 1 and top 5 accuracies on the ImageNet reference dataset, while being smaller and significantly faster than network architectures achieving comparably high accuracy rates on the same dataset [30]. The EfficientNet architecture has been recently developed and uses a compound scaling method to balance the width, depth, and resolution of a network. It has been successfully applied to histopathological image classification tasks previously [31].

The interpretability and explainability of trained models are important, as non-explainable results of a “black box” algorithm are not acceptable in the medical field, specifically in the diagnostic context for patients or interpretation of research data. In this regard, SmoothGrad is a very innovative and state-of-the-art technique and has an advantage over older methods that take a gradient of a class prediction neuron with respect to the input pixels, because it has much less background noise [32]. Thus, it can be appreciated that single cells or even specific cellular structures such as nuclei or the cytoplasm are important for the algorithmic classification decision. However, this is not a definite proof that the identified features are causally linked to a specific class, but it provides strong evidence that morphological patterns, and not unrelated image characteristics (e.g., a specific type of scanner, or staining characteristics), are the major reason for a particular classification result. Indeed, we could demonstrate that cells and nuclear size and shape are the key features for our model to separate the different histological classes. In this regard, our approach is well in line with the currently applied histomorphological criteria, e.g., to separate normal pancreatic ducts from PanIN based on cellular size and cellular shape, as well as the size and localization of the nuclei.

The application of our model to whole slides allows rapidly identifying anatomical tissue structures in pancreatic tissue and to localize areas with a high tumor content. This may result in a faster and focused review of tissue sections in the routine diagnostic, as regions of special interest are already highlighted. In addition, this approach provides a second control mechanism, as the tissue areas of interest are tagged. Moreover, tissue slides and areas with a high tumor cell content are automatically selected for further analysis and research.

Since we had established the algorithm on punch cores of a tissue microarray, with a core diameter of 1 mm, applications are not only limited to full HE sections but may also include small clinical fine needle biopsies, generated by ultrasound (EUS-FNBs). An EUS-FNB of PDAC often contains only a tiny amount of tumor cells, intermixed with large amounts of stroma and other tissue components and thus represents a diagnostic challenge for pathologists. In these cases, the proposed algorithm may help to solidify the diagnosis. Due to the good performance in discriminating exocrine from endocrine cells, also a value for tumor typing is possible, especially in cases where, due to a lack of enough tumor cells, immunohistochemistry for further characterization is not possible. However, the latter will require further adaptations to the algorithm.

Our model achieved high classification accuracies but had weaknesses in several classes. However, most of the misclassifications can be explained. Firstly, in many cases, more than one class is represented in one image (e.g., endocrine parenchyma adjacent to exocrine parenchyma, normal pancreatic ducts surrounded by exocrine parenchyma, or single tumor cells in tumor-associated fibrosis). Secondly, the group of non-tumor fibrosis, found in chronic pancreatitis or as scaffold tissue, and tumor-associated fibrosis is poorly defined and sometimes hard to distinguish. As pancreatic adenocarcinoma has a prominent stromal component, which may be important to understand the particularly aggressive nature of this cancer type, we labeled the fibrotic stroma as a separate class in addition to non-tumor fibrosis, knowing that both classes morphologically exhibit overlapping features. Thirdly, the group of PanIN low-grade and PanIN high-grade is prone to some degree of subjectivity, and it is possible that other observers may have labeled single images differently. Fourthly, PanIN high-grade and adenocarcinoma can only be distinguished by the identification of stromal invasion and loss of the lobular tissue architecture. When tilting the images, although great care was taken with the annotations, some images may not exhibit stroma. Thus, the differentiation feature might not always be represented, and it is impossible to distinguish both classes in these images. Last, lymph node metastases of pancreatic adenocarcinoma may induce a prominent stromal reaction and consume the non-neoplastic lymph node tissue. Therefore, adjacent non-infiltrated lymph node tissue might not be well represented on the images, which may explain the higher degree of misclassifications in the classes “lymph node metastasis” compared to “pancreatic adenocarcinoma”. All these issues, however, can be solved by individual cross-checking of the images by a pathologist.

The limitations of our study are mainly due to the number of included classes and the process for hyperparameter tuning. Herein, we trained our model on 201 cases and a total of 11 classes. In this regard, it is clear that only a fraction of the entire possible morphological spectrum with all inter- and even intra-individual variations of normal tissue structures, PanIN, and different growth patterns of PDAC will be included. Our model was trained to detect only one malignant pancreatic tumor (PDAC). Therefore, it cannot be expected that the algorithm will reliably identify intrapancreatic metastases from other primary tumors, neuroendocrine neoplasms, solid pseudopapillary neoplasms, the different cystic neoplasms of the pancreas, or the rare pancreatic tumors, which all were not trained in the current study. As already mentioned, PDAC is characterized by a prominent stromal component and may grow in a single-cell fashion. Thus, the small number of tumor cells per image patch may be a limiting factor for training, and the minimal number of tumor cells per image patch which is needed for the best reliable results is currently not clear. Based on the abovementioned statements, the application of deep learning approaches to classify pancreatic tissue must always be conducted under the supervision of a trained pathologist to avoid misclassifications and potentially harmful consequences for patients, although it represents a useful supplement.

## 4. Materials and Methods

### 4.1. Patient Cohorts, TMA Construction, and Scanning of Tissue Slides

We used a TMA consisting of a total of 113 patients (Institute of Pathology, University Medical Center of Mainz). TMA construction was conducted as described previously [33,34]. Diagnoses were made according to the 2019 World Health Organization Classification of Tumors of the digestive system [35]. Pancreatic TMA slides were scanned at 400× magnification using a slide scanner (NanoZoomer 2.0-HT, Hamamatsu Photonics K.K., Hamamatsu, Japan) as previously described [36].

Moreover, image patches were available from 88 patients acquired from other TMAs derived from the Tissue Biobank of the National Center for Tumor Diseases Heidelberg (NCT Heidelberg) with a different slide scanner (Aperio SC2, Leica Biosystems, Nussloch, Germany). The same slide scanner was used to scan representative whole slides from four other patients. The study was approved by the Institutional Review Board of Heidelberg University (IRB; #S315/20). The tissues of both cohorts were taken from resection specimens of patients who underwent primary surgery for pancreatic cancer.

**Figure 6:**
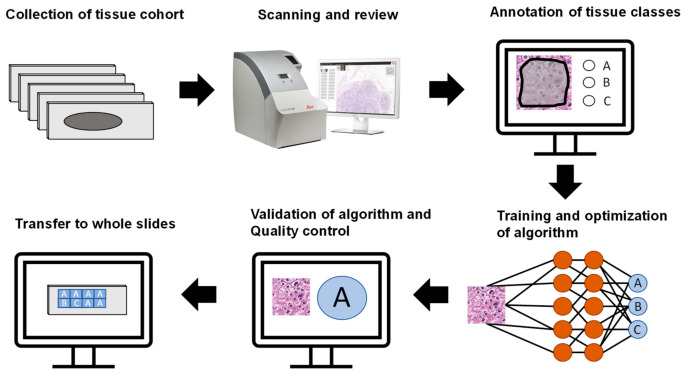
General workflow of the study. After collection of the tissue cohort, slides were scanned and reviewed. Tissue classes (A, B, C in this example) were annotated, and an algorithm was trained and optimized to classify these categories. The algorithm was validated, quality control was implemented, and the algorithm was applied to whole slides.

To ensure reliable results, patients from the TMAs were randomly separated into training (120, 60%), validation (41, 20%), and test sets (40, 20%). All image patches from a patient were used in the respective set. These subsets were not changed during the analyses. The general workflow of the study is outlined in Figure 6.

### 4.2. Annotation of Regions of Interest and Image Patch Extraction

**Figure 7:**
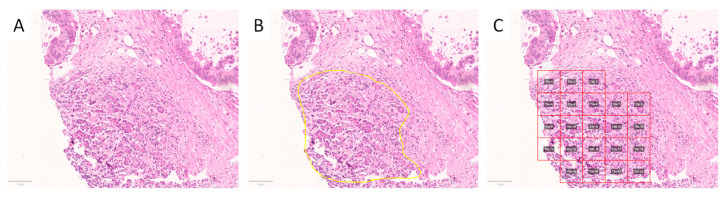
Annotation and generation of image patches. Representative tissue microarray core of a pancreatic specimen without (**A**) and with annotation of exocrine parenchyma ((**B**), yellow outline), as well as after image patches’ creation ((**C**), red squares). The image patches were subsequently saved as.png files. Scale bars: 100 µm.

**Figure 8:**
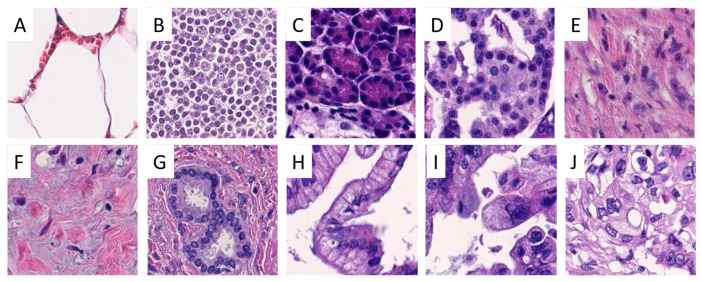
Examples of image patches from annotated areas. One representative image patch from fat (**A**), lymph node (**B**), exocrine (**C**) and endocrine parenchyma (**D**), stroma (**E**), tumor-associated stroma (**F**), normal pancreatic ducts (**G**), pancreatic intraepithelial neoplasia low-grade (**H**) and high-grade (**I**), and pancreatic ductal adenocarcinoma (**J**) is shown. Lymph node metastasis from pancreatic adenocarcinoma is not displayed. Magnification: each image 100 × 100 µm (219 × 219 px).

Scanned slides were imported into QuPath (v.0.1.2, University of Edinburgh, Edinburgh, UK). Areas of interest were annotated by a pathologist (A.K. and M.K.). Patches 100 × 100 µm (219 × 219 px) in size were generated within QuPath, and the tumor-associated image patches were exported to the local hard drive. Representative areas of interest and generated/extracted image patches are displayed in Figure 7 and Figure 8.

### 4.3. Hardware and Software 

For training and predicting with our models, we used the BwForCluster MLS&WISO Production nodes that feature the Nvidia GeForce RTX 2080Ti. We used a single GPU. Further, we applied singularity to adopt (v.3.6.4) and run (v.3.0.1) the TensorFlow 2.3.1-gpu docker container for training and predicting with our models. We added R (v.4.0.3) with packages dplyr (v.1.0.4), tidyr (v.1.1.2), tibble (v.3.0.6), config (v.0.3.1), readbitmap (v.0.1.5), data.tree (v.1.0.0), jsonlite (v.1.7.2), and jpeg (v.0.1.8.1), as well as the python packages pandas (v.1.1.5), Pillow (v.8.1.0), scipy (v.1.5.4), tabulate (v.0.8.7), and tensorflow_addons (v.0.12.1), to the container. The SmoothGrad and whole slide heatmaps were generated on a Lenovo P1 Gen 2 running Windows 10 with a conda (v.4.9.1) environment containing tensorflow (v.2.3.1), albumentations (v.0.5.2), and tf-explain (v.0.3.0). We adopted the tf-explain package code such that the resulting heatmaps were normalized to (0,1), and the maximum gradient per channel was used as described [37].

### 4.4. Model Training and Optimization 

We used models from the EfficientNet family for our analysis. The EfficientNet family is composed of multiple models (from B0 to B7) that are each a scaled version of the baseline model B0. The models are scaled by the compound scaling method introduced in [31]. With compound scaling, each consecutive model increases in network width, depth, and image resolution by a set of fixed scaling coefficients. This form of scaling utilizes the idea that the network width, depth, and image resolution seem to exhibit a certain relationship [31]. A model with fewer trainable weights can be trained using less resources, and its inference is faster [31]. In this study, we investigated the performance of the B0, B1, and B2 architectures. The non-trainable model parameters (such as dropout) provided in the tensorflow implementation of EfficientNet models were used without modification. The batch size was chosen as the maximal allowed valued (in the sequence of 2^n^, n ∈ N) given the available GPU memory. This batch size usually becomes smaller when scaling up an EfficientNet: the image resolution increases and the model itself becomes bigger due to the additional weights. We used the Adam optimizer with a learning rate that was selected for each model as follows: Models were trained for 50 epochs (each a pass of the full training data) with various learning rates roughly in the range of 1e-05 to 1e-06. Then, the best performing learning rate was chosen, and the respective model was trained further until there seemed to be no performance gain. Performance was visually evaluated by the achieved validation and training accuracies, the amount of overfitting (difference between training and validation accuracies), and the smoothness of the accuracy curves. The models with the highest validation accuracy for each class of EfficientNet models (B0, B1, B2) were compared, and the overall best performing one was used to classify the test set. Image augmentation was applied as described [38]. We used the basic, morphology, brightness and contrast, and hue/saturation/value augmentations. We adjusted some parameters by visually inspecting the generated augmented patches as follows. We used fixed instead of variable values for the elastic transform (α = 80, σ = 9); however, we applied the transformation with 50% chance only. We increased the range of sigma of the Gaussian blur from (0, 0.1) to (0, 1). For hue and saturation, we used a middle ground between the two settings HSV-light and HSV-strong: (−0.3, 0.3).

### 4.5. Model Explainability and Classification of Whole Slide Images

To further investigate if the network was learning the correct features, we applied SmoothGrad [32] to a selection of image patches. SmoothGrad produced heatmaps within an image patch that indicate the importance of certain pixels towards the prediction of a certain class. We used a noise level of 0.5% and a sample size of 50.

Moreover, we applied the model with the highest BACC to novel whole slide images. For the whole slide, image patches (395 × 395 px) with information on their localization were produced, extracted, classified, and reassembled to create heatmaps with information on the localization of the respective classes. To reduce the computational resources, empty (containing no tissue) vs. non-empty patches were classified as follows: An overview image of the whole slide was produced (largest dimension 1024 px) which was segmented into foreground and background using Otsu’s method [39]. If more than half of the pixels in the area of a patch of the overview image were considered background, the patch was classified as empty.

## 5. Conclusions

We used a CNN to automatically classify different anatomical structures including PDAC in pancreatic tissue specimens. This approach can be used as a supplementary method in the routine diagnostic setting to highlight worrisome structures which may need further review by pathologists. Moreover, it is a very helpful tool for tissue-based molecular research, especially for studying tumor heterogeneity and inflammation.

## Figures and Tables

**Table 1:** Characteristics of included PDAC/PanIN patients.

Gender, Female/Male		56:55
Median Age, Years (Range)		69.9 (37–82)
Tumor Stage		
pT (*n* = 107)	pT1	10
	pT2	67
	pT3	29
	pT4	1
	n.a.	6
pN (*n* = 111)	pN0	37
	pN1	41
	pN2	33
	n.a.	2
Grade (*n* = 111)	G1	5
	G2	62
	G3	43
	G4	1
	n.a.	2
Tumor size (*n* = 107)	≤1	0
	>1, ≤2	10
	>2, ≤4	68
	>4	29
	n.a.	6

**Table 2:** Number of extracted image patches per group.

Class	Set		
	Training	Validation	Test
Endocrine Islands	593	202	1990
Exocrine Islands	11,384	4828	5548
Fatty Tissue	2526	1241	1058
Lymph Node Metastasis	2017	743	502
Non-Tumor Fibrosis	3455	1111	2111
Normal Ducts	878	293	467
Pancreatic Adenocarcinoma	8169	3396	4950
PanIN High-Grade	1350	437	598
PanIN Low-Grade	1410	365	837
Tumor-Associated Stroma	3981	1753	1847
Tumor-Free Pancreatic Lymph Node	7840	1950	1335

**Table 3:** Trained EfficientNet models with training and validation accuracies.

Model			Accuracy	
Arch.	Learning Rate	Batch Size	Training	Validation
B2	0.00001	64	0.83	0.82
B1	0.00001	64	0.83	0.81
B0	0.000005	128	0.77	0.80

**Table 4:** BACC for various PQC thresholds and non-aggregated as well as aggregated classes.

(Values in %)	0	0.5	0.6	0.7	0.8	0.9
BACC non-aggregated	65.63	67.21	68.78	70.62	71.87	73.2
BACC aggregated	76.69	78.88	80.77	82.85	86.39	92.12
Below threshold	0	8.98	18.66	29.3	40.67	53.59

## Data Availability

Data are available from the corresponding authors upon reasonable request.

## Supplementary Materials

The following are available online at https://www.mdpi.com/article/10.3390/ijms22105385/s1, Figure S1: Validation and test non-aggregated confusion matrices for all classes for the B2 EfficientNet model and various quality control (QC) limits. Table S1.
