# Supplementary material for: Deep Learning in Pancreatic Tissue: Identification of Anatomical Structures, Pancreatic Intraepithelial Neoplasia, and Ductal Adenocarcinoma
Source: Int J Mol Sci. 2021 May 20;22(10):5385. doi: 10.3390/ijms22105385 (PMC8160892; doi:10.3390/ijms22105385)
Supplement: Supplementary file 1 [file ijms-22-05385-s001.zip › ijms-1183457-supplementary.pdf]

Suppl. Figure 1.: Validation and test non-aggregated confusion matrices for all classes for the B2 EfficientNet model and various quality control (QC) limits.

Test set

QC 0.5:

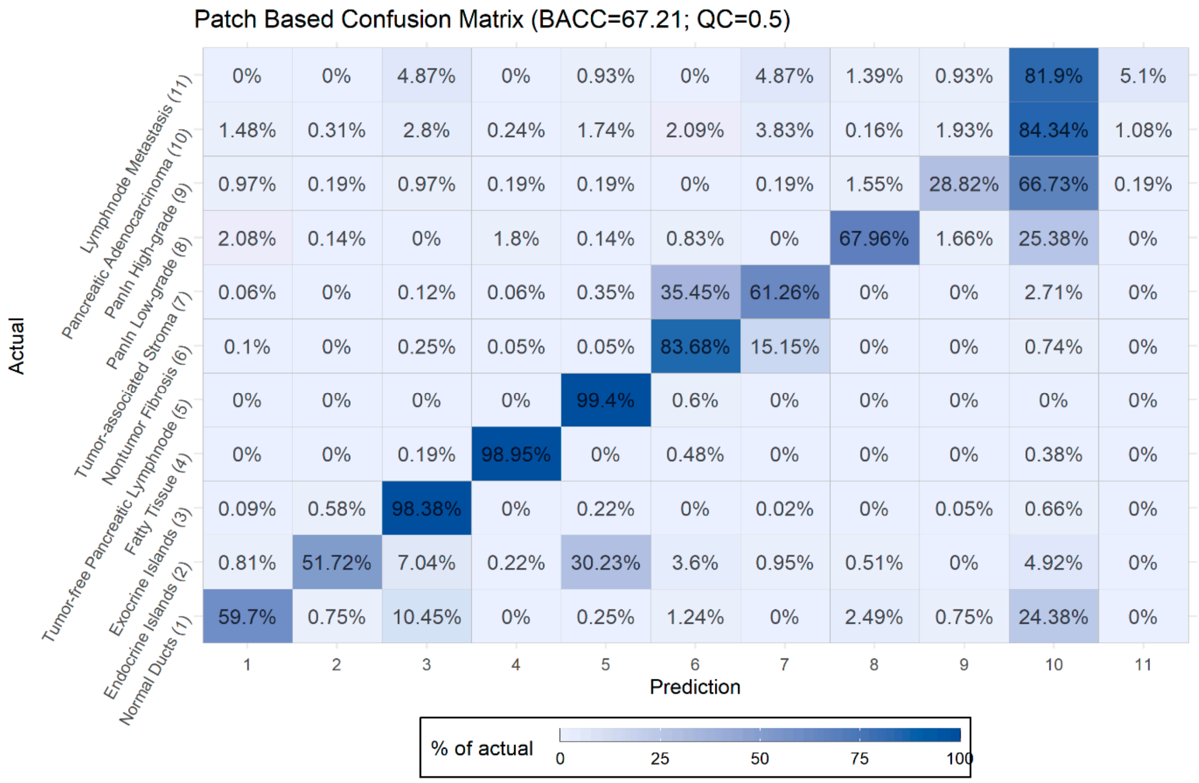

QC 0.6

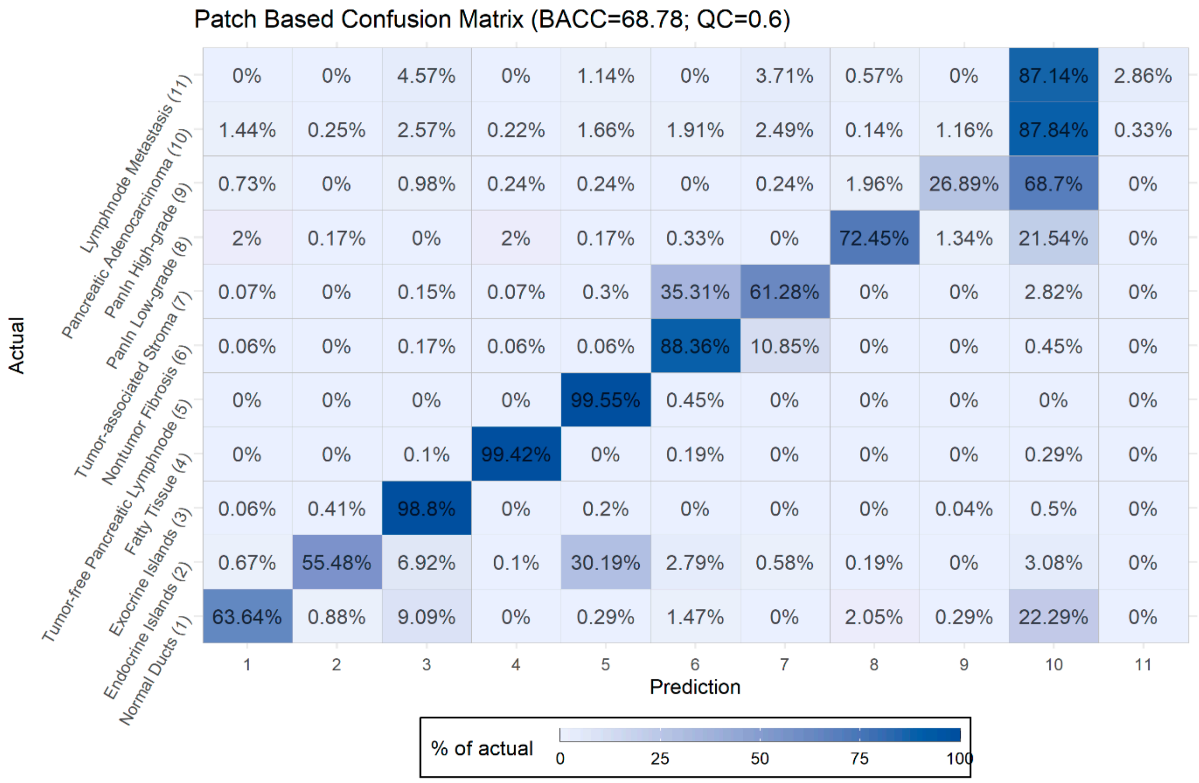

QC 0.7

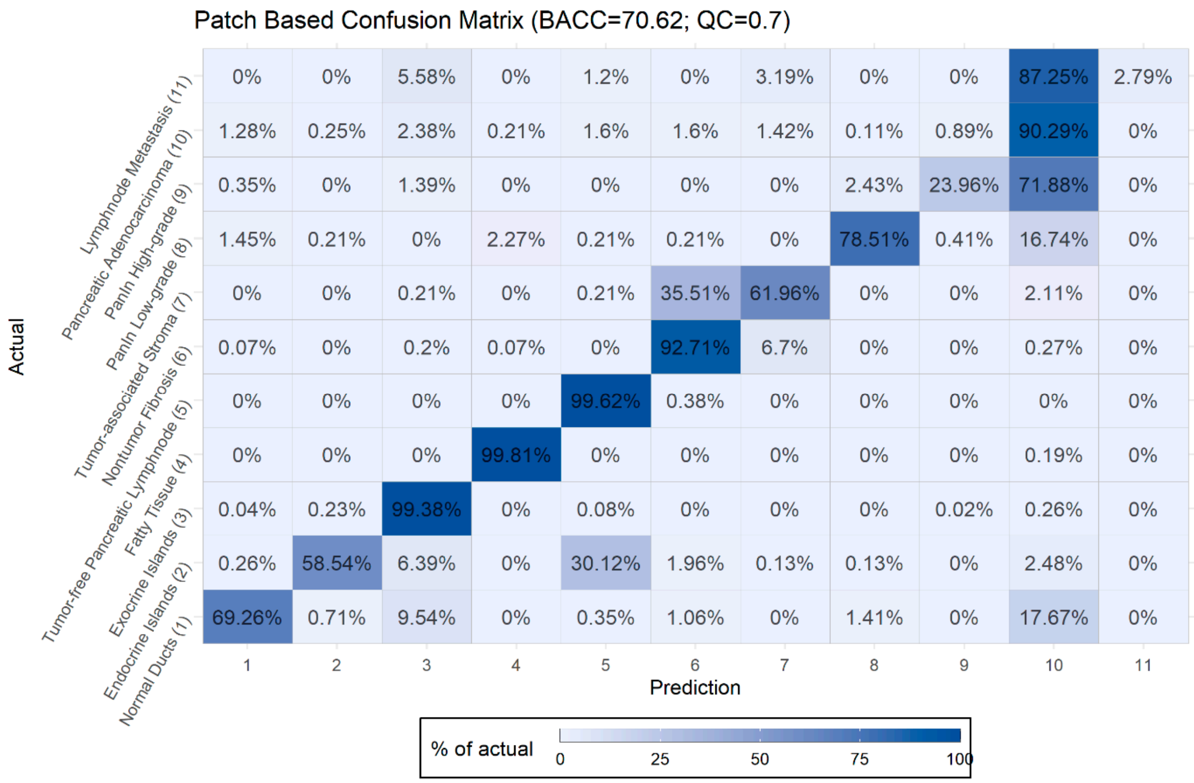

QC 0.8

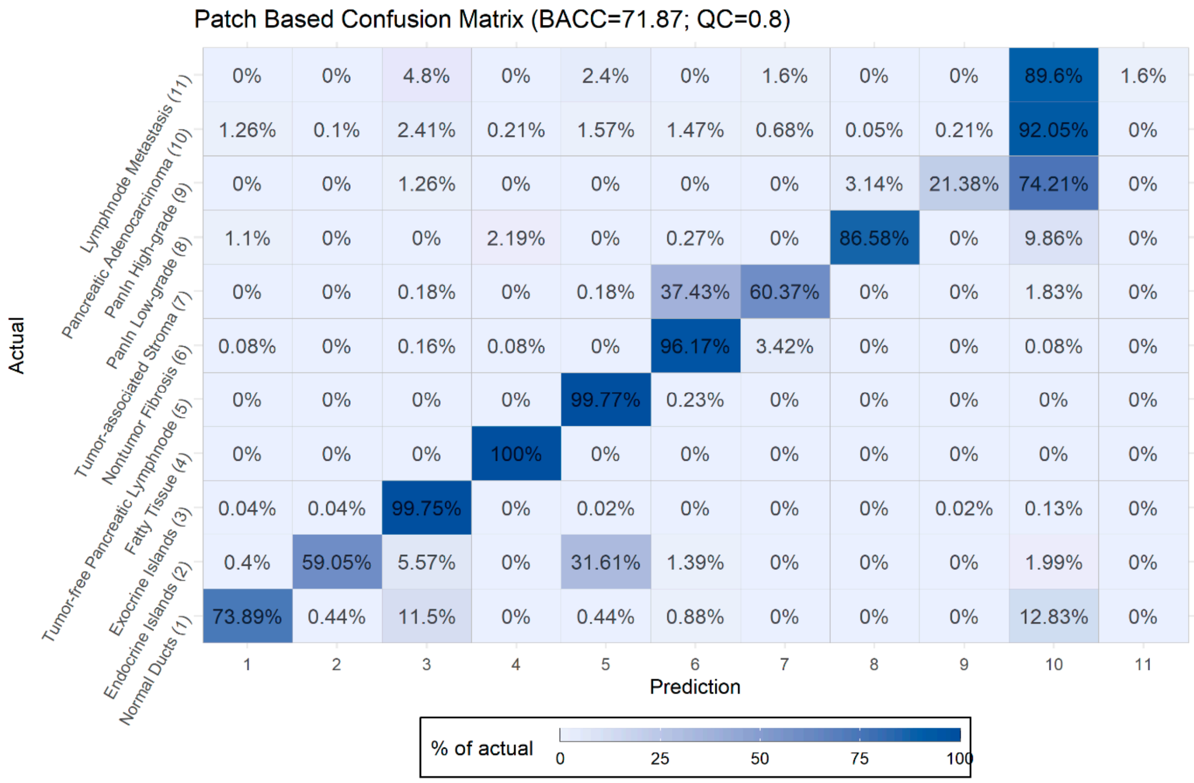

QC 0.9

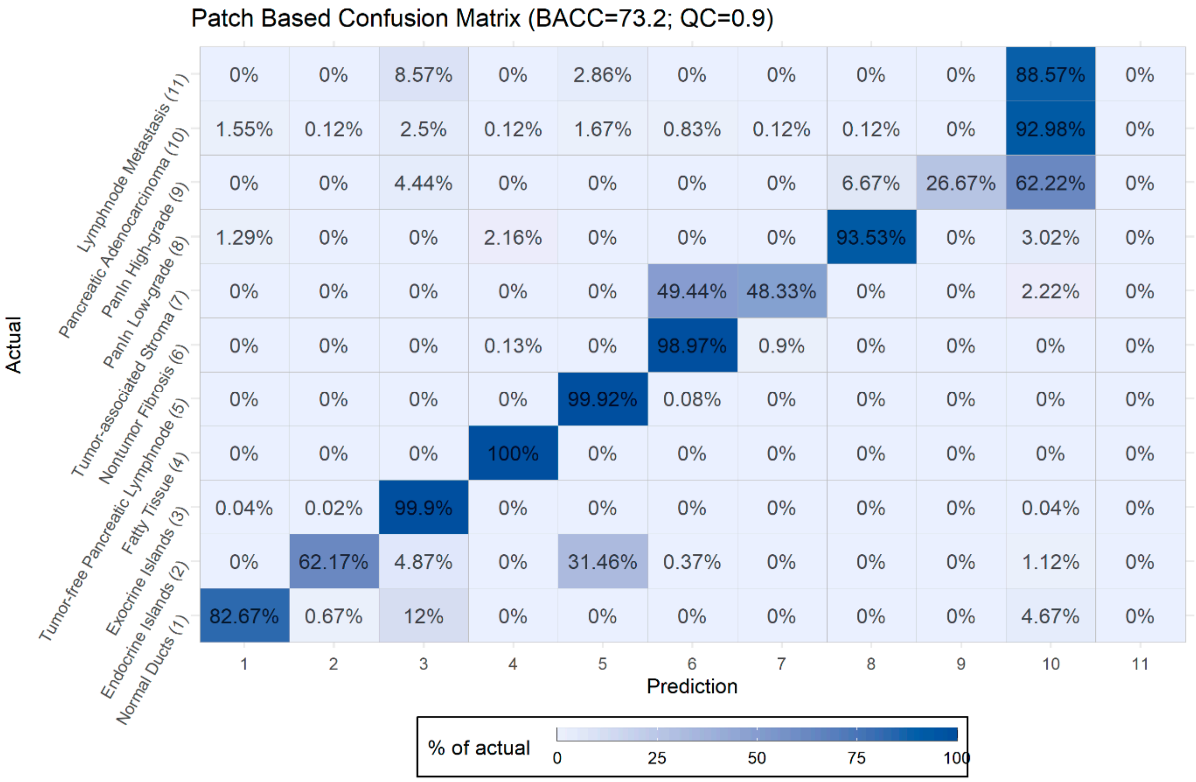

Patch Based Confusion Matrix (BACC=73.2; QC=0.9)
Actual
Lymphnode Metastasis (11)
Pancreatic Adenocarcinoma (10)
PanIn High-grade (9)
PanIn Low-grade (8)
Tumor-associated Stroma (7)
Nontumor Fibrosis (6)
Tumor-free Pancreatic Lymphnode (5)
Fatty Tissue (4)
Exocrine Islands (3)
Endocrine Islands (2)
Normal Ducts (1)
0% 0% 8.57% 0% 2.86% 0% 0% 0% 0% 88.57% 0%
1.55% 0.12% 2.5% 0.12% 1.67% 0.83% 0.12% 0.12% 0% 92.98% 0%
0% 0% 4.44% 0% 0% 0% 0% 6.67% 26.67% 62.22% 0%
1.29% 0% 0% 2.16% 0% 0% 0% 93.53% 0% 3.02% 0%
0% 0% 0% 0% 0% 49.44% 48.33% 0% 0% 2.22% 0%
0% 0% 0% 0.13% 0% 98.97% 0.9% 0% 0% 0% 0%
0% 0% 0% 0% 99.92% 0.08% 0% 0% 0% 0% 0%
0% 0% 0% 100% 0% 0% 0% 0% 0% 0% 0%
0.04% 0.02% 99.9% 0% 0% 0% 0% 0% 0% 0.04% 0%
0% 62.17% 4.87% 0% 31.46% 0.37% 0% 0% 0% 1.12% 0%
82.67% 0.67% 12% 0% 0% 0% 0% 0% 0% 4.67% 0%
1 2 3 4 5 6 7 8 9 10 11
Prediction
% of actual
0 25 50 75 100

Validation set

QC 0.5

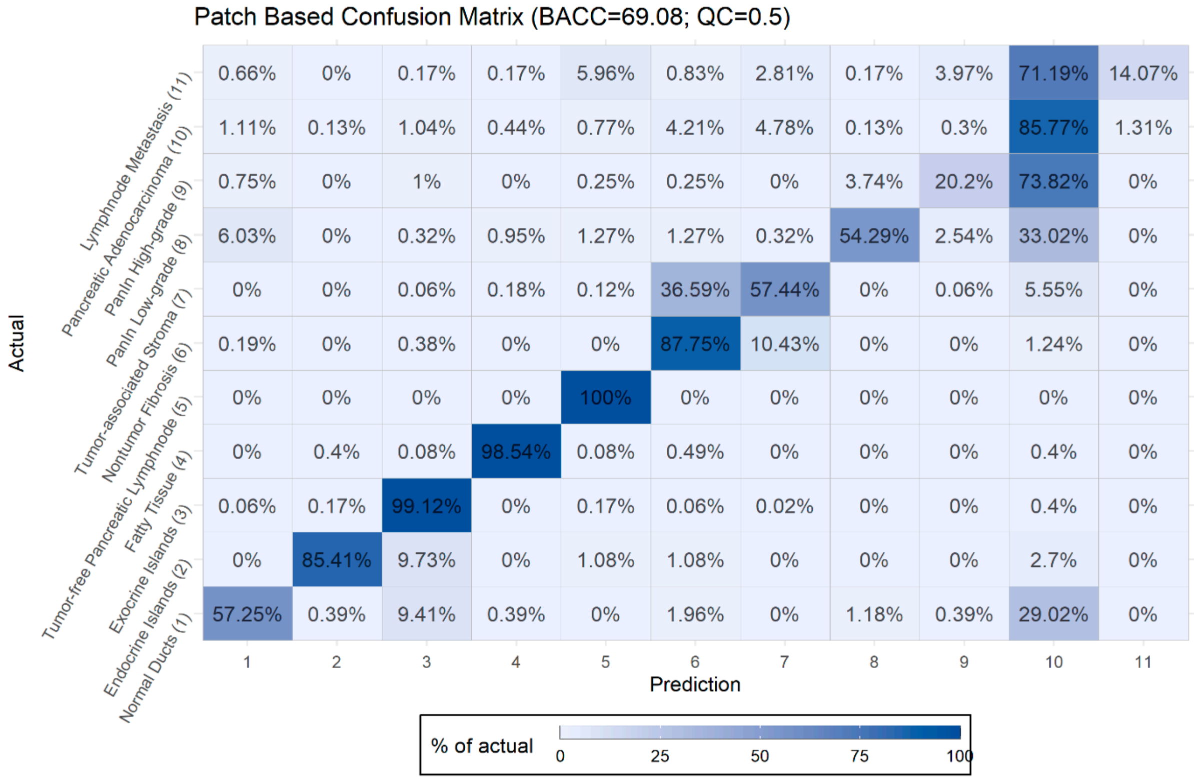

QC 0.6

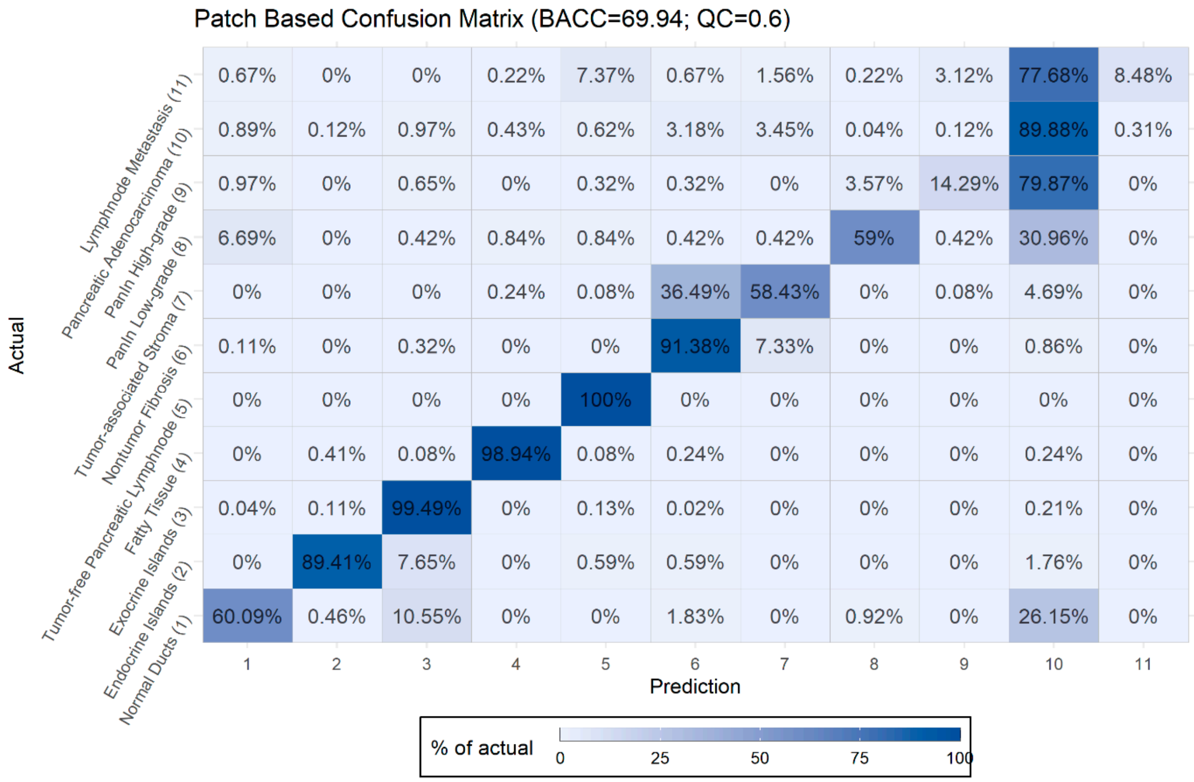

QC 0.7

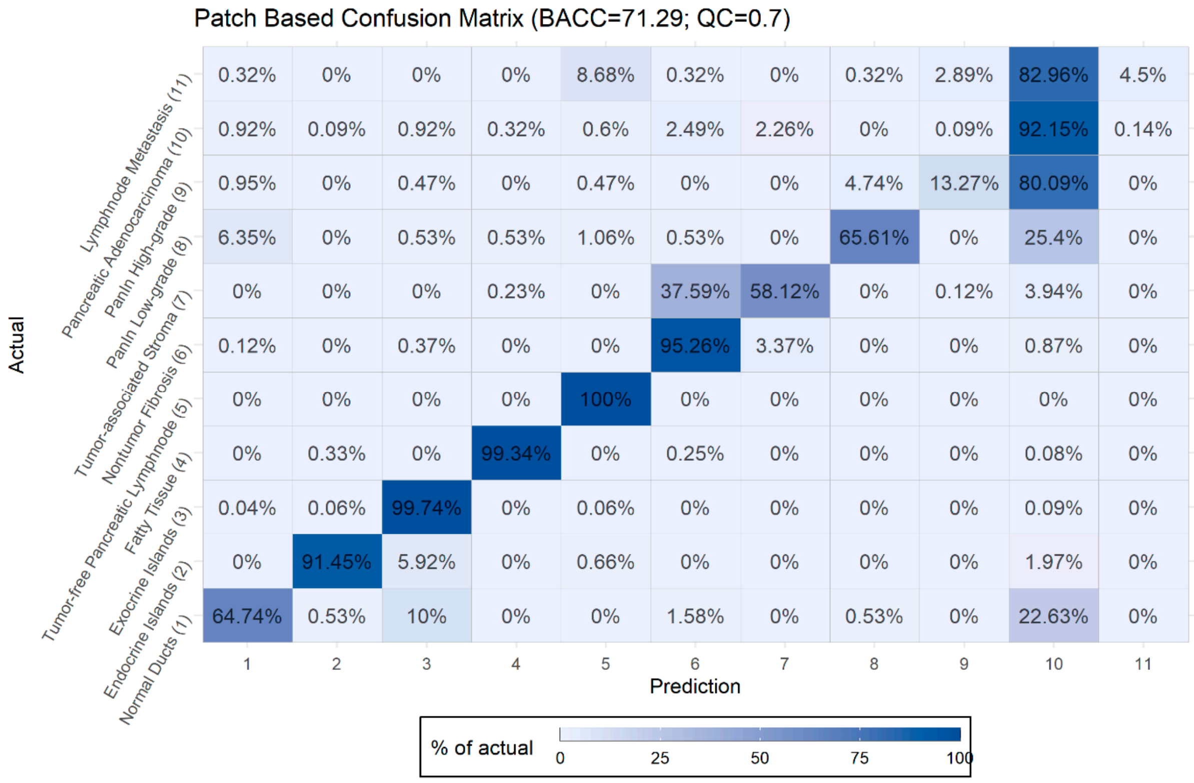
Patch Based Confusion Matrix (BACC=71.29; QC=0.7)
Actual
Lymphnode Metastasis (11)
Pancreatic Adenocarcinoma (10)
PanIn High-grade (9)
PanIn Low-grade (8)
Tumor-associated Stroma (7)
Nontumor Fibrosis (6)
Tumor-free Pancreatic Lymphnode (5)
Fatty Tissue (4)
Exocrine Islands (3)
Endocrine Islands (2)
Normal Ducts (1)
0.32% 0% 0% 0% 8.68% 0.32% 0% 0.32% 2.89% 82.96% 4.5%
0.92% 0.09% 0.92% 0.32% 0.6% 2.49% 2.26% 0% 0.09% 92.15% 0.14%
0.95% 0% 0.47% 0% 0.47% 0% 0% 4.74% 13.27% 80.09% 0%
6.35% 0% 0.53% 0.53% 1.06% 0.53% 0% 65.61% 0% 25.4% 0%
0% 0% 0% 0.23% 0% 37.59% 58.12% 0% 0.12% 3.94% 0%
0.12% 0% 0.37% 0% 0% 95.26% 3.37% 0% 0% 0.87% 0%
0% 0% 0% 0% 100% 0% 0% 0% 0% 0% 0%
0% 0.33% 0% 99.34% 0% 0.25% 0% 0% 0% 0.08% 0%
0.04% 0.06% 99.74% 0% 0.06% 0% 0% 0% 0% 0.09% 0%
0% 91.45% 5.92% 0% 0.66% 0% 0% 0% 0% 1.97% 0%
64.74% 0.53% 10% 0% 0% 1.58% 0% 0.53% 0% 22.63% 0%
1 2 3 4 5 6 7 8 9 10 11
Prediction
% of actual
0 25 50 75 100

QC 0.8

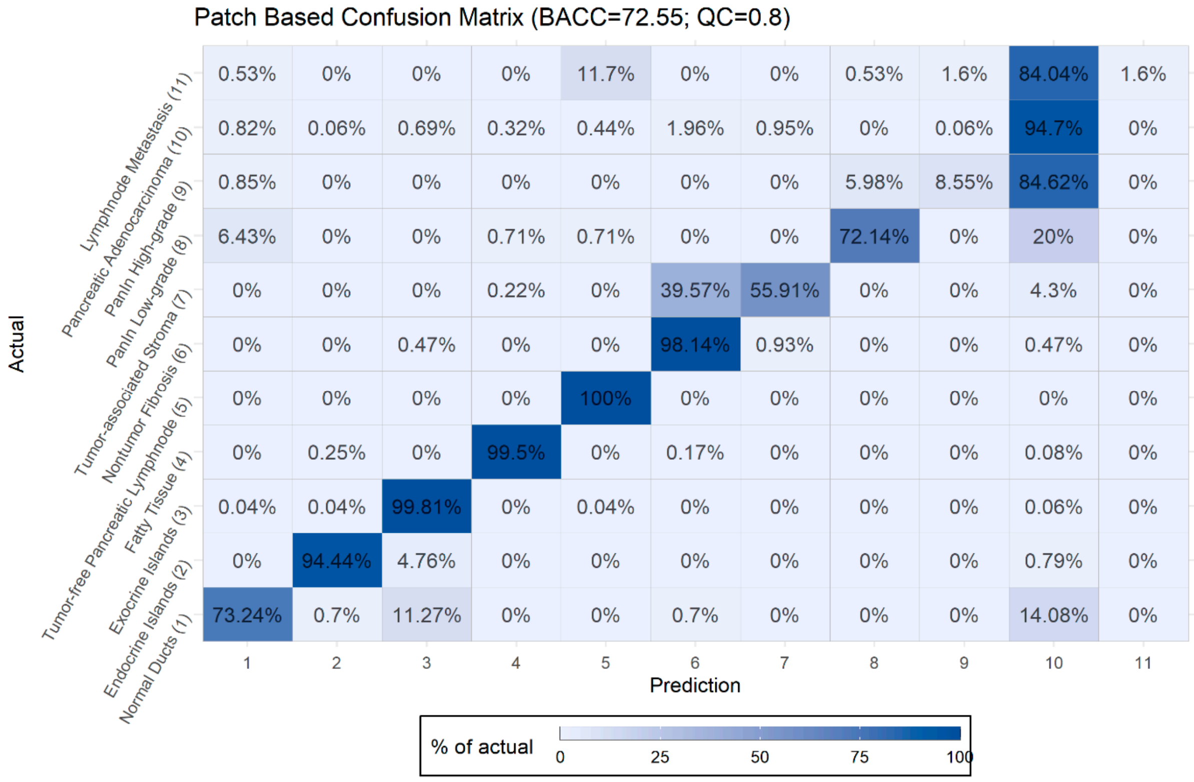
Patch Based Confusion Matrix (BACC=72.55; QC=0.8)
Actual
Lymphnode Metastasis (11)
Pancreatic Adenocarcinoma (10)
PanIn High-grade (9)
PanIn Low-grade (8)
Tumor-associated Stroma (7)
Nontumor Fibrosis (6)
Tumor-free Pancreatic Lymphnode (5)
Fatty Tissue (4)
Exocrine Islands (3)
Endocrine Islands (2)
Normal Ducts (1)
0.53% 0% 0% 0% 11.7% 0% 0% 0.53% 1.6% 84.04% 1.6%
0.82% 0.06% 0.69% 0.32% 0.44% 1.96% 0.95% 0% 0.06% 94.7% 0%
0.85% 0% 0% 0% 0% 0% 0% 5.98% 8.55% 84.62% 0%
6.43% 0% 0% 0.71% 0.71% 0% 0% 72.14% 0% 20% 0%
0% 0% 0% 0.22% 0% 39.57% 55.91% 0% 0% 4.3% 0%
0% 0% 0.47% 0% 0% 98.14% 0.93% 0% 0% 0.47% 0%
0% 0% 0% 0% 100% 0% 0% 0% 0% 0% 0%
0% 0.25% 0% 99.5% 0% 0.17% 0% 0% 0% 0.08% 0%
0.04% 0.04% 99.81% 0% 0.04% 0% 0% 0% 0% 0.06% 0%
0% 94.44% 4.76% 0% 0% 0% 0% 0% 0% 0.79% 0%
73.24% 0.7% 11.27% 0% 0% 0.7% 0% 0% 0% 14.08% 0%
1 2 3 4 5 6 7 8 9 10 11
Prediction
% of actual
0 25 50 75 100

QC 0.9

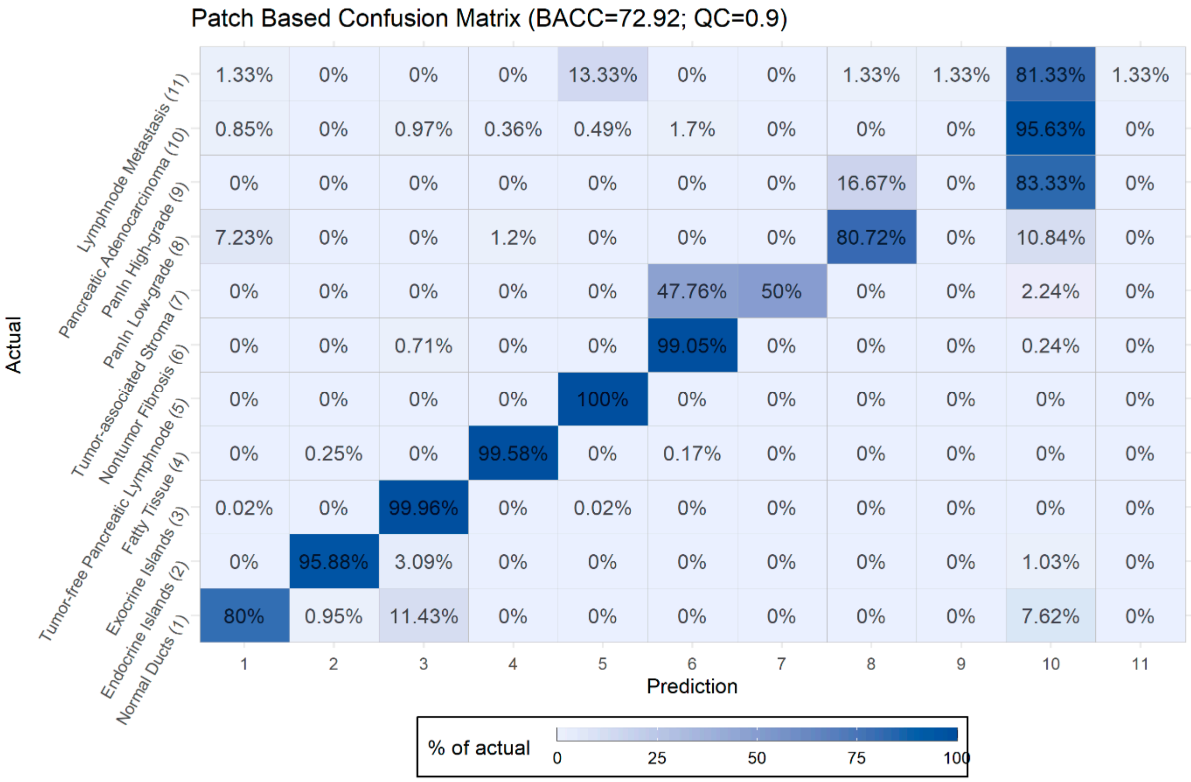
Patch Based Confusion Matrix (BACC=72.92; QC=0.9)
Actual
Lymphnode Metastasis (11)
Pancreatic Adenocarcinoma (10)
PanIn High-grade (9)
PanIn Low-grade (8)
Tumor-associated Stroma (7)
Nontumor Fibrosis (6)
Tumor-free Pancreatic Lymphnode (5)
Fatty Tissue (4)
Exocrine Islands (3)
Endocrine Islands (2)
Normal Ducts (1)
1.33% 0% 0% 0% 13.33% 0% 0% 1.33% 1.33% 81.33% 1.33%
0.85% 0% 0.97% 0.36% 0.49% 1.7% 0% 0% 0% 95.63% 0%
0% 0% 0% 0% 0% 0% 0% 16.67% 0% 83.33% 0%
7.23% 0% 0% 1.2% 0% 0% 0% 80.72% 0% 10.84% 0%
0% 0% 0% 0% 0% 47.76% 50% 0% 0% 2.24% 0%
0% 0% 0.71% 0% 0% 99.05% 0% 0% 0% 0.24% 0%
0% 0% 0% 0% 100% 0% 0% 0% 0% 0% 0%
0% 0.25% 0% 99.58% 0% 0.17% 0% 0% 0% 0% 0%
0.02% 0% 99.96% 0% 0.02% 0% 0% 0% 0% 0% 0%
0% 95.88% 3.09% 0% 0% 0% 0% 0% 0% 1.03% 0%
80% 0.95% 11.43% 0% 0% 0% 0% 0% 0% 7.62% 0%
1 2 3 4 5 6 7 8 9 10 11
Prediction
% of actual
0 25 50 75 100

Suppl. Table 1

	Class	Slide_1_n	Slide_1_perc	Slide_2_n	Slide_2_perc	Slide_3_n	Slide_3_perc	Slide_4_n	Slide_4_perc
1	Fatty Tissue	437	1,28	1097	2,04	1140	2,35	393	1,1
2	Tumor-free Pancreatic Lymphnode	315	0,92	2092	3,89	927	1,91	5642	15,8
3	Exocrine Islands	7965	23,34	2395	4,46	19	0,04	524	1,47
4	Endocrine Islands	96	0,28	348	0,65	171	0,35	104	0,29
5	Nontumor Fibrosis	1362	3,99	9662	17,99	6010	12,37	4404	12,33
6	Tumor-associated Stroma	166	0,49	2227	4,15	7488	15,41	2171	6,08
7	Normal Ducts	101	0,3	182	0,34	19	0,04	42	0,12
8	PanIN Low-grade	59	0,17	242	0,45	30	0,06	65	0,18
9	PanIN High-grade	10	0,03	18	0,03	48	0,1	22	0,06
10	Pancreatic Adenocarcinoma	211	0,62	1495	2,78	2341	4,82	1929	5,4
11	Lymph node Metastasis	0	0	1	0	11	0,02	4	0,01
12	Empty	22200	65,05	31946	59,48	28676	59,01	17586	49,24
13	Multiple predictions	0	0	9	0,02	1	0	1	0
14	QC Failed	1206	3,53	1996	3,72	1713	3,53	2830	7,92
